# Over prescription of antibiotics for adult pharyngitis is prevalent in developing countries but can be reduced using McIsaac modification of Centor scores: a cross-sectional study

**DOI:** 10.1186/1471-2466-12-70

**Published:** 2012-11-24

**Authors:** Amber Hanif Palla, Rafeeq Alam Khan, Anwar H Gilani, Fawziah Marra

**Affiliations:** 1 Department of Pharmacology, University of Karachi, Karachi, Pakistan; 2 Department of Biological and Biomedical Sciences, Aga Khan University Medical College, Karachi, Pakistan; 3 Department of Basic Medical Sciences, College of Medicine, King Saud bin Abdul-Aziz University for Health Sciences, King Abdul-Aziz Medical City, Jeddah, 21423, KSA; 4 Faculty of Pharmaceutical Sciences, University of British Columbia, Vancouver, Canada

**Keywords:** Antibiotics, Pharyngitis, McIsaac-modified Centor score, Antibiotic prescribing, Pakistan

## Abstract

**Background:**

Although *Group A beta hemolytic streptococcus (GABHS)* can cause bacterial pharyngitis, the most common etiology is viral; despite this viral etiology, antibiotics are commonly prescribed for this infection in industrialized countries. We investigated the prevalence of *GABHS* in adult pharyngitis patients from lower socioeconomic settings in Karachi, Pakistan, how often antibiotics are prescribed for pharyngitis and if appropriate agents were used in a developing world setting. Finally, we wanted to see the usefulness of modified McIsaac scores in predicting positive cultures.

**Methods:**

Adult patients were recruited from three local hospital outpatient dispensaries (OPDs). All patients aged 14–65 years who were suspected of having bacterial pharyngitis had throat swabs taken. Laboratory results for *GABHS* pharyngitis were then compared with their prescriptions. Appropriateness (using the World Health Organization’s definition) and type of antibiotic prescribed were assessed.

**Results:**

Of 137 patients**,** 30 patients each were studied for scores of 0, 1, 2 and 3; 17 patients were studied for score 4. Although 6 (4.4%) patients were *GABHS*+, for a prevalence of 43.8 per 1000 population, antibiotics were prescribed to 135 patients (98.5%). Of these, only 11.1% received appropriate antibiotics while 88.9**%** received inappropriate antibiotics**.** Penicillins were prescribed most (34.1%), especially amoxicillin/clavulanate; followed by macrolides (31.1%), especially the second-generation agents, and fluoroquinolones (14.8%). McIsaac scores were found to be 100% sensitive and 68.7% specific, giving a positive predictive value (PPV) of 12.7% and a negative predictive value (NPV) of 100%.

**Conclusions:**

Antibiotics were prescribed irrationally to adult pharyngitis patients, as most cultures were negative for bacterial infection. McIsaac modification of Centor scores related directly to culture results. We would therefore highly recommend its use to help family physicians make treatment decisions for adult pharyngitis patients.

## Background

*Group A beta hemolytic Streptococcus (GABHS)* is commonly implicated in bacterial pharyngitis [1]. Starting treatment with antibiotics for *GABHS* infection, within the first 24–48 hours of illness, when a bacterial cause is highly suspected, has been found to decrease duration of symptoms, such as sore throat, fever and adenopathy by approximately one day [2], and prevent complications of *GABHS* pharyngitis, particularly rheumatic fever and rheumatic heart disease [3]. However, the majority of pharyngitis cases in adults are of viral etiology [4]; only 5–15% of cases suffer from bacterial pathogens that require prompt antibiotic treatment [5,6].

The World Health Organization (WHO) defines an appropriate prescription as “administration of the right drug indicated for the disease, in the right dose, through an appropriate route of administration, for the right duration” [7]. When these criteria are not fulfilled, the prescription is considered inappropriate. Inappropriate antibiotic prescriptions for treatment of pharyngitis have contributed to the emergence of resistant strains of oropharyngeal human flora [8] which in turn, have increased morbidity, mortality, and health-care costs [9]. Approximately three quarters of pharyngitis patients have received inappropriate antibiotic prescriptions, by receiving antibiotics for viral infections or otherwise not adhering to the WHO definition [10-12].

First-line agents for treatment of bacterial pharyngitis include penicillin, ampicillin or amoxicillin.^13^ Alternative options include erythromycin (especially in patients with a non-life-threatening allergy to penicillin) and first-generation cephalosporins (CG) [1]. Both erythromycin and cephalosporins are also considered reasonable alternatives to penicillin in patients who fail to respond to penicillin or continue to become re-infected following penicillin therapy [13-15]. As *GABHS* is the most important pathogen causing infection, fluoroquinolones, and sulfamethoxazole/trimethoprim that do not cover Gram-positive pathogens very well are not recommended. Although amoxicillin-clavulanate, clarithromycin, azithromycin and second-generation cephalosporins work very well against *GABHS* infection, they are considered third-line alternatives due to their broader spectrum of action and potential for causing resistance.

Throat swab and culture is the gold standard for diagnosis of pharyngitis. A rapid antigen detection test (RADT) can also give relatively specific diagnosis in a physician’s office. Although a WHO technical report states that there is less possibility of false-positive results with RADT, RADT kits vary in sensitivity, which ranges from 31–95%. Therefore, RADT cannot be substituted for standard blood agar cultures [3]. Because antibiotics treatment should occur fairly promptly, diagnosis of pharyngitis is often based on clinical symptoms; throat swabs are not always taken [16]. Thus to improve the diagnostic criteria, several scoring systems have been developed to predict, on a clinical basis, whether patients have bacterial or viral pharyngitis [17,18].

Among the many devised clinical scores, the Centor criteria are reliable predictors of *GABHS* pharyngitis. They include evaluating patients for tonsillar exudates, tender anterior cervical lymphadenopathy or lymphadenitis, absence of cough, and history of fever (oral temperature greater than 38.3°C; 101°F) [19]. More recently, the Centor score was modified by incorporating patient's age, which allows the physician to place patients in low-, moderate-, or high-risk groups. The use of the McIsaac Modified Centor score has helped in decreasing inappropriate antibiotic use by almost 88% [18].

Several guidelines have been published on diagnosis and treatment of streptococcal pharyngitis in adults; however, not all are in agreement. The American College of Physicians’ (ACP) guideline endorsed by Centre for Disease Control (CDC), American Academy of Family Physicians and the American Society of Internal Medicine, recommend that patients with low Centor scores of 0 or 1 (i.e., low risk for streptococcal pharyngitis) do not require any testing or treatment with antibiotics. For patients with Centor scores of 2 or, 3, the guidelines suggest using a RADT, which would give a sensitivity of > 80% for accurate diagnosis of *GABHS* infection, and prescribing antibiotics to patients with positive tests [20,21]. Empirical treatment with antibiotics is recommended for patients with Centor scores of 3 or 4 [22]. However, practice guidelines issued by the American Heart Association [23], American Academy of Pediatrics [24], and Infectious Diseases Society of America (IDSA) [25] recommend microbiologic confirmation by throat culture or RADT to diagnose all adults with pharyngitis prior to antibiotic prescribing, regardless of their Centor scores. IDSA and others are of the opinion that if prestigious organizations like AAFP and CDC endorse the option of not culturing at all for any given score, it would be unlikely that physicians would opt for either RADT or culture [26].

These concerns were addressed by McIsaac who pointed out that missed infections are not due solely to score approaches, as physicians do not obtain a throat swab for every case of sore throat [27]. Therefore, treatment decisions based on clinical judgment would already miss 50% of *GABHS* infections, while 20–40% of the larger number of non-*GABHS* sore throat presentations would be identified as needing antibiotics [19,28].

Given the controversy in clinical guidelines and not knowing how physicians in Pakistan generally treat their adult pharyngitis patients, we investigated whether antibiotics are prescribed appropriately for primary treatment of pharyngitis within a developing world setting and under low socio-economic conditions. We also evaluated the sensitivity and specificity of McIsaac Modified Centor scores in predicting *GABHS* pharyngitis in our patient populations. Finally, we evaluated whether the choice of antibiotic to treat pharyngitis was appropriate, in terms of antibiotic class, dose, and duration of therapy. Our study did not address use of antibiotics for secondary prevention of pharyngitis or asymptomatic carrier state.

## Methods

### Study setting

The study was conducted in three tertiary care hospitals—the Jamal Noor hospital, Zubaida Medical Centre and the Civil Hospital, within two different areas of Karachi (Dhoraji and Bander Road) where outpatient dispensaries (OPD) were available. The clinical ethics committees of all three hospitals gave their approval. Before starting the study, educational sessions were set up at the clinics to describe the study and consent process to clinic nurses and physicians. Informed consent was obtained from all study participants by the clinic physician.

### Patient recruitment

All patients between the ages of 14 and 65 years of age, suspected of having bacterial pharyngitis were asked to participate in the study between March and October 2005. Patients who were younger than 14 years, immunocompromised (i.e., had autoimmune diseases including HIV/AIDS or were on immunosuppressive agents), or had been on antibiotics 24–48 hours before were not included in the study.

Patients who agreed to participate were categorized according to the McIsaac Modified Centor System into scores 0, 1, 2, 3 and 4 [27]. One point was assigned to each of the following symptoms: tonsillar exudates, tender anterior cervical lymphadenopathy or lymphadenitis, absence of cough, and history of fever (oral temperature greater than 38.3°C; 101°F), and one point was deducted if the patient was older than 45 years.

### Microbiology

A throat swab was collected by the clinic physician using a sterile swab from the posterior pharynx, tonsils and/or inflamed areas. These swabs were transported to the microbiological laboratory facility of Zubaida Medical Center, which is an ISO 2001 certified laboratory and follows the Monica Cheesbrough “District laboratory practice in tropical countries” guidelines [29] for specimen testing, and the Clinical and Laboratory Standards Institute (CLSI) guidelines [30] to perform the cultures.

Each specimen was cultured on a sheep blood agar plate and a chocolate agar plate in anaerobic environment at 35°–37°C for 18–24 hours before reading for *GABHS*, and then in CO_2_ rich atmosphere 35°C for 24 hours. A colony grown on blood agar plate and chocolate agar was taken, and streaked on the nutrient agar plate. A bacitracin disc, and penicillin, ampicillin, amoxicillin, amoxicillin-clavulanate, cephradine, clarithromycin and erythromycin, were then placed on the plate and were incubated for 24 hours. Based on zones of inhibition, they were graded as sensitive, intermediate or resistant. Testing methodology was same for all patients.

### Definitions

Inappropriate treatment was defined as per the WHO, which suggests that administration of the right drug indicated for the disease, in the right dose, through an appropriate route of administration, for the right duration [6]. Thus, both infected (*GABHS*+) patients who did not receive antibiotics, and uninfected (*GABHS*–) patients who did receive antibiotic prescriptions were considered to receive inappropriate treatment.

A second aspect of appropriate antibiotic prescription was whether the choice of antibiotic for *GABHS*+ pharyngitis was from a recommended class, in the right dose and for the right duration. When evaluating a physician’s choice of antibiotics, we assumed that all patients who received antibiotics were infected; prescriptions were then considered appropriate if they prescribed an accurate dose and duration of first-line agents or second-line alternative agents. Prescriptions were considered inappropriate if patients received inappropriate doses or duration of first- or second-line agents, or third- line agents or antibiotics that are not recommended for pharyngitis infection.

First-line antibiotics included penicillin (oral penicillin V 500 mg every 8 hours for 10 days; benzyl penicillin 0.6–1.2 million units IM once; oral ampicillin 500 mg every 6 hours for 10 days; oral amoxicillin 500 mg every 8 hours for 10 days) [20]. Appropriate alternative second-line agents included cephalexin (500 mg every 12 hours for 10 days) and other first-generation cephalosporins, cefaclor (500 mg every 8 hours orally for 10 days), a second-generation cephalosporin [31] and erythromycin for penicillin-allergic patients (250 mg p.o. every 6 hours or 500 mg every 12 or 6 hours for 10 days).

Third-line agents were considered inappropriate, because they are too broad-spectrum, or do not have adequate activity. Broad-spectrum agents included amoxicillin-clavulanate (500–875 mg orally every 12 hours for 10 days), second-generation macrolides such as clarithromycin (250 mg orally every 12 hours for 5 days), azithromycin (500 mg orally on day 1; 250 mg on days 2–5 for 5 days) and roxithromycin (150 mg orally every 12 hours or 300 mg once daily for 10 days), broader-spectrum second-generation cephalosporins like cefuroxime (250 mg or 500 mg every 12 hours for 5–10 days) and third-generation cephalosporins like cefixime (400 mg orally daily for 5 days). Use of erythromycin for non-penicillin allergic patients was also considered inappropriate. Finally, antibiotics not recommended for Gram-negative pathogens were also considered to be inappropriate or third-line agents; these included fluoroquinolones (e.g., ciprofloxacin, levofloxacin and ofloxacin); sulfonamides such as sulfamethoxazole/trimethoprim and tetracyclines (e.g., doxycycline, minocycline, tetracycline, and oxytetracycline).

### Study outcomes and statistics

The primary outcome was the prevalence of *GABHS* infection in the adult pharyngitis patients from a low socioeconomic setting. Secondary outcomes included 1) number of prescriptions used for adult pharyngitis; 2) appropriateness of prescribed antibiotics; and 3) diagnostic accuracy of the Modified Centor criteria.

By comparing patients’ culture results, we could determine the prevalence of infection. To determine whether patients received appropriate or inappropriate prescriptions, culture results were compared with antibiotic prescriptions. Sensitivity, specificity, positive predictive and negative predictive value of the McIsaac score approach was determined by ratios of false positives, true positives, false negatives and true negatives.

## Results

Table 1 shows the demographics of the study population. Of the 137 patients, the average age of the study population was 26 years old while the median age was 23 years. There were more males than females who presented with symptoms of pharyngitis (66% male vs 34% females). Thirty patients each with scores 0, 1, 2 and 3, and 17 patients with score 4 were evaluated for bacterial pharyngitis (N= 137); of these patients, only 6 were *GABHS*+ but 135 were treated with antibiotics. Thus, the prevalence of bacterial pharyngitis in our population was 43.8 per 1000 population. Penicillin was the most frequently prescribed antibiotic class (34.1%). Within this class, the majority of prescriptions were for amoxicillin-clavulanate (26.6%), a broad-spectrum penicillin. Approximately 15% of the prescriptions were for cephalosporins (14.8%), with the third-generation cephalosporins accounting for 9.6% of the usage, second-generation cephalosporins accounting for 3.7%, and first-generation cephalosporins accounting for 1.4% of total antibiotic usage. Macrolides (31.1%), quinolones (14.8%), sulfonamide (3.0%), tetracyclines (2.2%) were also prescribed for patients.

**Table 1:** Patient demographics

**Variables**	**N (%)**
**Total number of patients in study**	137
Males, N (%)	91 (66.4%)
Females, N (%)	46 (33.5%)
Mean age ± SD	26 ± 10.71
Median age ± SD	23 ± 10.71
**Antibiotics prescribed by physicians**	
Penicillin	46 (34.1%)
Macrolides	42 (31.1%)
Cephalosporins	20 (14.8%)
Quinolones	20 (14.8%)
Sulfonamide/Trimethoprim	4 (3.0%)
Tetracyclines	3 (2.2%)

Antibiotics were prescribed inappropriately to adult pharyngitis patients; we saw no association between antibiotic use and culture confirmation results (*P* = 0.75). When cross tabulated, of the patients who were given antibiotics, only 4% patients were *GABHS*+; 96% of patients were *GABHS*– (Table 2).

**Table 2:** Appropriateness of antibiotic prescribing when compared to culture results

**Culture from throat swab**	**Antibiotic prescribed**		**Total**
	**Yes**	**No**	
Positive, N (%)	6	0	6 (4.4%)
Negative, N (%)	129	2	131 (95.6%)
Total, N (%)	135	2	137 (100%)

A total of 135 patients received antibiotics, but only 15/135 (11.1%) received appropriate antibiotics; 120/135 (88.9%) who received inappropriate antibiotics, not recommended by current guidelines. Table 3 shows the breakdown of the antibiotics received by the patients. Only 8/135 (5.8%) of the patients received first-line agents for treatment of pharyngitis; most patients received second-line agents (7/135; 5.2%) and third-line agents (120/135; 88.9%). Of the inappropriate antibiotics prescribed, macrolides (42/135; 31.1%) were prescribed most, including erythromycin for non-penicillin allergic patients, clarithromycin and roxithromycin; followed by broad-spectrum penicillin; amoxicillin-clavulanate (36/135; 26.7%), the third-generation cephalosporin cefixime (13/135; 9.6%) and fluoroquinolones (20/135; 14.8%).

**Table 3:** Antibiotics prescribed by physicians for adult pharyngitis patients

**APPROPRIATE ANTIBIOTICS**		
**Class**	**Name**	**Numbers (%)**
**Appropriate first-line agents**		
Penicillin	Ampicillin	3 (2.2%)
	Amoxicillin	5 (3.7%)
Total		8 (5.8%)
**Appropriate alternative second-line agents**		
First-generation cephalosporins	Cefadroxil	1 (0.7%)
	Cephradine	1 (0.7%)
Second-generation cephalosporins	Cefaclor	5 (3.7%)
Total		7 (5.2%)
**NON-RECOMMENDED ANTIBIOTICS**		
**Inappropriate or third-line agents**		
Broad spectrum penicillins	Amoxicillin-Clavulanate	36 (26.7%)
	Ampicillin-Cloxacillin	2 (1.5%)
Macrolides	Erythromycin for patients not reported with penicillin allergy	14 (10.5%)
	Clarithromycin	27 (20.0%)
	Roxithromycin	1 (0.7%)
Third-generation cephalosporins	Cefixime	13 (9.6%)
Quinolones	Ciprofloxacin	2 (1.5%)
	Levofloxacin	18 (13.3%)
Sulfonamides	Sulfamethoxazole/trimethoprim	4 (3.0%)
Tetracyclines	Oxytetracycline	3 (2.2)
Total		120 (88.9%)

Antibiotics were prescribed by brand names to 96.2% of patients. Irrespective of which class of antibiotic was prescribed, only 45% (61/135) of patients were prescribed antibiotics at appropriate doses and durations, whereas 55% (74/135) received prescriptions for inappropriate doses and/or durations.

Table 4 shows an even distribution of patients in each of the 4 score groups (30 in each group), except for group 4 (17 patients). The McIsaac score system was found to be 100% sensitive and 68.7% specific, giving a positive predictive value (PPV) of 12.7% and a negative predictive value (NPV) of 100%.

**Table 4:** Cross tabulation of McIsaac modification of Centor score and culture

**McIsaac modification of Centor score**	**Culture positivity (N = 137)**		**Total**	**TN**^**3**^	**TP**^**3**^	**FP**^**3**^
Score 0	0 (0%)	30 (100%)	30	30	–	0
Score 1	0 (0%)	30 (100%)	30	30	–	0
Score 2	0 (0%)	30 (100%)	30	30	–	0
Score 3	1 (3.4%)	29 (96.6%)	30	0	1	29
Score 4	5 (29.4%)	12 (70.6%)	17	0	5	12
Sensitivity^4^	100% (95% CI: 0.5–1)					
Specificity^4^	68.7% (95% CI: 0.54–0.7)					
PPV^3,1^	12.7% (95% CI: 0.05–0.26)					
NPV^3,2^	100% (95% CI: 0.9–1)					

^1^ Yes = Number of patients GABHS+ in culture / Total number of patients with respective score.

^2^ No = Number of patients not found GABHS+ in culture.

^3^ TN = True negative; TP = True positive; FP = False positive; PPV = positive predictive value; NPV = negative predictive value.

^4^ Sensitivity and specificity of scores 0 to 4.

## Discussion

This is the first study to look at appropriate antibiotic use for adult pharyngitis in a developing world setting. Our study showed that much antibiotic use is unnecessary for patients who have pharyngitis—probably because most such patients have viral, rather than bacterial, infections. In fact, only 4.3% of the patients in our sample were found to be *GABHS*+; the rest were likely viral infections.

Furthermore, even when antibiotics are used, physicians are prescribing broader class agents for treatment, such as amoxicillin/clavulanate or macrolides, rather than using the simple narrow-spectrum agents such as penicillin or amoxicillin/ampicillin; even when the antibiotic choice was appropriate, doses and durations of those antibiotics mostly were not. Although benzathine penicillin is recommended by the WHO, we did not see its usage in our study. Our investigation did not interrogate physicians’ motives; however, we speculate that they preferred the ease and lower cost of oral antibiotics to benzathine penicillin, which costs more and requires intramuscular injection.

Although our findings are similar to those of other studies conducted in western countries, the enormity of the problem seems to be much larger in Pakistan due to the large numbers of patients seeking medical attention for pharyngitis [32,33]. Many factors could have contributed to inappropriate prescribing of antibiotics for pharyngitis. Quality of health care is constrained by costs in developing countries like Pakistan. Therefore, for a common ailment like sore throat, a costly throat swab and culture is not a routine practice. Furthermore, RADT is not an option in our setting because of its unavailability and high cost. Finally, patients generally expect to get the maximum benefit from a physician visit so that they do not have to pay the cost of the next visit. As a result, physicians are burdened with patients’ expectation of providing all care during the patient’s first and only visit. The results of our study would be generalizable to other low income countries such as India, Afghanistan and Bangladesh.

Our study showed that it would be useful for clinicians in Pakistan to use the McIsaac Modified Centor score as it is not costly, and is sensitive and specific enough to reduce unnecessary antibiotic prescriptions. Based on our study, we recommend that the score-only approach would save antibiotics prescriptions for most patients, as most adult patients with pharyngitis had scores of 0, 1 or 2. Although without a RADT test option, more antibiotics would be prescribed for patients with a score of 3 or 4, this percentage is better than the percentage of patients getting inappropriate antibiotics without clinical score-based screening at all. This modified Centor clinical prediction rule holds more importance in our setting because RADT is not widely available in our country; given their high cost and varied sensitivity (31–95%) of the different RADT kits, their use will be limited [3]. During the course of our study, we saw that affordability of RADT testing was the major concern in this setting, which resulted in almost 99% of patients being prescribed antibiotics. Following a score approach would significantly reduce unnecessary antibiotics prescriptions.

Our study implies that, as with developed countries, clinicians in low socioeconomic countries need further education on appropriate antibiotics to use for *GABHS* infection, particularly the proper dose and duration of those antibiotics and possible repercussions of inappropriately prescribed antibiotics. Education should emphasize the higher prevalence of viral etiology, use of the McIsaac modified Centor scoring system, and published guidelines on treatment of pharyngitis [8,14]. This is particularly important as India and Pakistan have been found to be among the initial sites of extremely resistant organisms such as *Escherichia coli* and *Klebsiella pneumonia* with the carbapenem-resistance gene *bla*NDM-1, [34] which may have resulted from uncontrolled and excessive use of antibiotics in these countries. Inappropriate use of antibiotics in developing regions has consequences for everyone; microbes have no country. Sensitivity to this issue needs to be renewed by creating awareness that should be well supported by data.

This study has several limitations. This patient sample was very small with representation of few selected settings where the influx of patients is from lower- and middle-class families. A larger sample with patients from different areas of Karachi in future studies would offer better-supported conclusions about city-wide prescribing practices, and about the prevalence of *GABHS* infection in adults. The McIsaac–Centor score has been validated for use in industrialized countries, where prevalence of rheumatic heart disease is < 1 per 1000 population, compared to >10 per 1000 in Pakistan /Asia [35]. Thus, using the McIsaac modified score as a routine clinical tool may also mean that more cases of rheumatic fever are being prevented, given the higher prevalence of rheumatic fever in Pakistan compared to the industrialized nations.

## Conclusions

In Pakistan, antibiotics are prescribed for most cases of adult pharyngitis when in fact, the majority of the cultures are negative for bacterial infection. Furthermore, it was alarming for us to discover the high use of second generation macrolides and cephalosporins rather than the recommended narrower spectrum agents. McIsaac modification of Centor score directly related to culture results. We therefore highly recommend its use to help family physicians evaluate appropriate use of antibiotics.

## Competing interests

The authors declare that they have no competing interests.

## Authors’ contributions

Ms. AHP designed the study, obtained ethics approval, collected and analyzed the data, created initial manuscript draft and subsequent revisions. Dr. FM assisted with statistical analysis, helped with the initial manuscript draft and revised all subsequent drafts. Drs RAK and AHG commented on study design and all manuscript drafts. All authors read and approved the final manuscript.

## Pre-publication history

The pre-publication history for this paper can be accessed here:

http://www.biomedcentral.com/1471-2466/12/70/prepub
